# Metabolic Control of m^6^A RNA Modification

**DOI:** 10.3390/metabo11020080

**Published:** 2021-01-30

**Authors:** Joohwan Kim, Gina Lee

**Affiliations:** 1 Department of Microbiology and Molecular Genetics, University of California Irvine School of Medicine, Irvine, CA 92697, USA; juhwan828@gmail.com; 2 Department of Microbiology and Molecular Genetics, Chao Family Comprehensive Cancer Center, University of California Irvine School of Medicine, Irvine, CA 92697, USA

**Keywords:** *N*^6^-methyladenosine, m^6^A, RNA methylation, RNA chemical modification, RNA epitranscriptome, metabolites, nutrient signaling, metabolic pathways

## Abstract

Nutrients and metabolic pathways regulate cell growth and cell fate decisions via epigenetic modification of DNA and histones. Another key genetic material, RNA, also contains diverse chemical modifications. Among these, *N*^6^-methyladenosine (m^6^A) is the most prevalent and evolutionarily conserved RNA modification. It functions in various aspects of developmental and disease states, by controlling RNA metabolism, such as stability and translation. Similar to other epigenetic processes, m^6^A modification is regulated by specific enzymes, including writers (methyltransferases), erasers (demethylases), and readers (m^6^A-binding proteins). As this is a reversible enzymatic process, metabolites can directly influence the flux of this reaction by serving as substrates and/or allosteric regulators. In this review, we will discuss recent understanding of the regulation of m^6^A RNA modification by metabolites, nutrients, and cellular metabolic pathways.

## 1. Introduction

RNA plays an essential role in gene expression control. In addition to transferring genetic information from DNA to protein, RNA controls protein expression by providing messenger RNA (mRNA) for translation. mRNA is generated by the processing of nascent RNA, which involves the splicing of introns, 5′cap addition, and 3′ polyadenylation. In addition to these well-known RNA maturation processes, RNA also undergoes chemical modification at its bases and ribose rings [1,2]. The *N*^6^-adenosine methylation (m^6^A or *N*^6^-methyladenosine) is the most abundant mRNA internal modification. It was discovered in the 1970s [3,4] when other RNA processes were discovered, although follow up studies have lagged. Nearly three decades later, the identification of methyltransferases [5,6,7] and demethylases [8,9] proved that m^6^A modification is not a random event, but rather an enzyme-mediated selective process. In addition, transcriptome-wide sequencing of m^6^A-modified mRNAs revealed that m^6^A is enriched around the stop codon and deposited at a consensus motif [10,11]. These seminal studies reignited m^6^A research, which is now extended to various RNA species including long noncoding RNA (lncRNA) [12,13], ribosomal RNA (rRNA) [14,15], and small nuclear RNA (snRNA) [16,17], opening a new field of RNA epitranscriptomics. m^6^A modification alters RNA structure and RNA–protein interactions, which control RNA fates such as splicing [18], stability [19,20,21], localization [22], and translation efficiency [23,24], ultimately affecting protein expression. m^6^A-dependent gene expression plays crucial roles in normal development including embryogenesis, stem cell maintenance [25,26], and neurogenesis [27,28] and its dysregulation causes diseases such as cancer [29,30,31] and diabetes [32,33]. There are several comprehensive reviews about molecular biological and pathophysiological functions of m^6^A [34,35,36,37]. In this review, we discuss m^6^A RNA modification from a metabolic perspective.

## 2. Enzymes Involved in m^6^A RNA Modification

**Figure 1:**
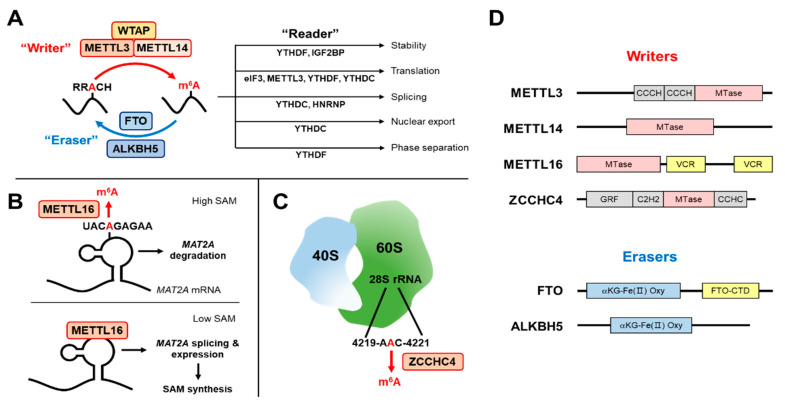
*N*^6^-methyladenosine (m^6^A) methylation process and its biological functions. (**A**) m^6^A writers (methyltransferase) methylate RNA in the adenine nucleobase of amino group at *N*^6^ position. The consensus motif of methyltransferase-like 3 (METTL3) is RRA*CH (R = A/G; A* = methylated A; H = A/C/U). Once m^6^A is deposited on RNA, m^6^A reader proteins are recruited and determine RNA fates, such as splicing, stability, and translation efficiency, which ultimately affect gene expression. m^6^A is removed from RNA through demethylation by eraser proteins. (**B**) METTL16 methylates stem-loop structure in 3’ untranslated region (UTR) of *S*-adenosyl methionine (SAM) synthase, *methionine adenosyltransferase 2A* (*MAT2A*). In SAM-repleted conditions, *MAT2A* is methylated and degraded. Oppositely, in SAM-depleted conditions, METTL16 induces splicing and expression of *MAT2A*. (**C**) Methylation of A4220 in 28S ribosomal RNA (rRNA) by zinc finger CCHC domain-containing protein 4 (ZCCHC4) promotes ribosome assembly and translation. (**D**) Domain composition of m^6^A enzymes. (Top, writers) m^6^A writers contain methyltransferase (MTase) domains. METTL3 contains Cys-Cys-Cys-His (CCCH) zinc finger motifs. METTL16 has two vertebrate conserved region (VCR) domains in C-terminus. ZCCHC4 possesses several zinc finger motifs, including Gly-Arg-Phe (GRF), Cys2-His2 (C2H2), and Cys-Cys-His-Cys (CCHC) domains. (Bottom, erasers) Fat mass and obesity-associated protein (FTO) and alkb homolog 5 (ALKBH5) contain αKG-Fe(II)-dependent dioxygenase domains conserved in dioxygenase family enzymes. WTAP, Wilms’ tumor 1-associated protein; eIF3, eukaryotic initiation factor 3; YTHDF, YTH domain family; YTHDC, YTH domain-containing protein; IGF2BP, insulin-like growth factor 2 mRNA-binding protein; HNRNP, heterogeneous nuclear ribonucleoproteins; FTO-CTD, FTO C-terminal domain.

The primary m^6^A writer complex is composed of methyltransferase-like 3 (METTL3), METTL14, and an adaptor protein, Wilms’ tumor 1-associating protein (WTAP) [7] (Figure 1A). Additional components of this complex are VIRMA/KIAA1429 [38], zinc finger CCCH-type containing 13 (ZC3H13) [39], and RNA binding motif protein 15 (RBM15) [13]. The writer complex methylates specific adenosine residues on mRNA and non-coding RNAs in RRA*CH consensus motif (R represents A or G; H represents A, C or U; A* is the methylated adenosine) [10,11].

In addition to the METTL3–METTL14 complex, there are several other classes of m^6^A RNA methyltransferases. METTL16 is primarily responsible for the methylation of snRNA and some mRNAs [40]. METTL16 targets a distinct consensus motif, UACA*GAGAA, in the RNA stem-loop structure [41,42] (Figure 1B). Different from the METTL3–METTL14 heterodimer complex, METTL16 functions as a homodimer [42].

18S and 28S rRNA methylations are catalyzed by METTL5-tRNA methyltransferase 112 (TRMT112) complex [14] and zinc finger CCHC domain-containing protein 4 (ZCCHC4) [15], respectively (Figure 1C). These proteins localize in the nucleolus where ribosome synthesis and maturation occur. In contrast to other methyltransferases, ZCCHC4 contains an autoinhibitory loop in the RNA-binding surface, which is opened upon 28S rRNA binding [43]. This sort of mechanism may determine substrate RNA specificity among various m^6^A methyltransferases.

Once adenosine is methylated, a variety of m^6^A binding proteins (readers) are recruited (Figure 1A). These include YT521-B homology domain family proteins (YTHDF and YTHDC) [44], heterogeneous nuclear ribonucleoproteins (HNRNP) [45], and insulin-like growth factor 2 mRNA-binding protein (IGF2BP) families [20]. These proteins control the fate of target RNAs, such as folding into secondary structures [45], splicing [18], nuclear export [22], liquid–liquid phase separation [46], stability [20,21], and translation [23,24].

On the other hand, demethylase enzymes (erasers) are responsible for removing m^6^A (Figure 1A). Potential m^6^A erasers are alkB homolog 5 (ALKBH5) and fat mass and obesity-associated (FTO, also known as ALKBH9) proteins, which belong to ALKB family of dioxygenases [8,9]. The discovery of these specific m^6^A processing proteins (i.e., writers, erasers and readers), provided evidence that m^6^A modification is a highly regulated, reversible cellular process.

## 3. Regulation of m^6^A Writers by SAM and SAH

**Figure 2:**
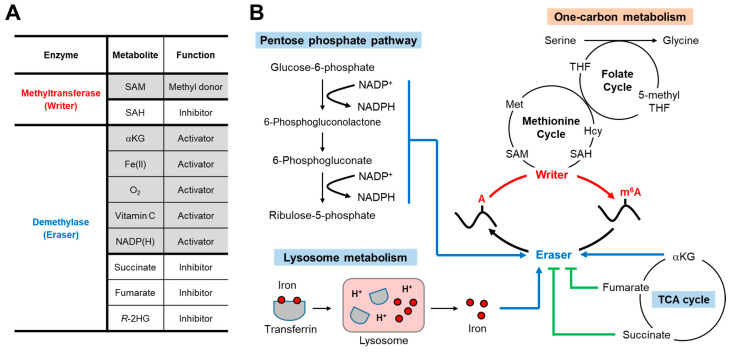
Potential interplay of m^6^A methylation with cellular metabolic pathways. (**A**) List of metabolites affecting activities of m^6^A writers and erasers. Grey, activators; white, inhibitors. (**B**) Schematic of metabolic pathways that can influence m^6^A methylation and demethylation processes. One-carbon metabolism produces SAM, a methyl donor of m^6^A modification. *S*-adenosyl homocysteine (SAH), the by-product of methylation, inhibits writer activity. On the other hand, the oxidative demethylation of m^6^A by erasers needs α-ketoglutarate (αKG), oxygen (O_2_), and iron [Fe(II)] as cofactors. Tricarboxylic acid (TCA) cycle produces co-factors that activate (αKG) or inhibit (fumarate and succinate) demethylase activity. Iron is delivered to cells as transferrin–iron complex, wherein low lysosomal pH causes release of free iron ions into the cytoplasm. Pentose phosphate pathway converts nicotinamide adenine dinucleotide phosphate (NADP^+^) to NADPH, another cofactor for demethylases. *R*-2HG, *R*-2-hydroxyglutrate; Met, methionine; THF, tetrahydrofolate; Hcy, homocysteine.

Similar to other typical enzymatic reactions, m^6^A writer-mediated methylation is dynamically regulated by substrates and products. *S*-adenosyl methionine (SAM/AdoMet) is a universal methyl donor for the cellular methylation processes (Figure 2A). Indeed, METTL3 was originally identified as a SAM-binding protein [5]. In cells, METTL3 forms a stable heterodimeric complex with METTL14. Even though both METTL3 and METTL14 contain methyltransferase domains (Figure 1D), the catalytic site of METTL14 lacks the SAM binding motif and only METTL3 contains enzymatic activity. Rather, METTL14 maintains METTL3–METTL14 complex stability and recruits RNA substrates for efficient m^6^A writing [47,48,49].

One-carbon metabolism, composed of folate and methionine cycles, is the metabolic pathway responsible for SAM production (Figure 2B). Two amino acids, serine and methionine, play key roles in providing carbons to this pathway. Serine provides a one-carbon unit to the tetrahydrofolate (THF) cofactor, generating methyl-THF. Then, another carbon acceptor, homocysteine, receives one-carbon from methyl-THF. On the other hand, methionine adenosyl transferase (MAT) produces SAM using methionine and adenosine 5′-triphosphate (ATP) as substrates. Finally, SAM provides a methyl group to adenosine on RNAs and becomes *S*-adenosyl homocysteine (SAH/AdoHcy). As is often the case with metabolic enzymes, the product of this methylation reaction, SAH, is a strong allosteric inhibitor of METTL3 methyltransferase activity [50] (Figure 2A).

It has been shown that the perturbation of cellular SAM levels affects DNA and histone methylation [51]. Interestingly, the *K*_m_ (substrate concentration at half maximum reaction rate) of SAM for METTL3 is much lower (~100 nM) than cellular SAM levels (>10 µM) [50,52], suggesting that METTL3 is constitutively active regardless of fluctuations in cellular SAM levels. Ironically, intracellular SAH levels (~5 µM) are higher than the IC_50_ (half maximal inhibitory concentration) of SAH for METTL3 (~1 µM) [50,52,53], suggesting that METTL3 can also be constitutively inhibited by high SAH levels. However, it is possible that the subcellular, local concentrations of SAM and SAH are likely different from their concentrations in total cell lysates. In addition, other binding proteins of SAM or SAH can change the levels of free SAM and SAH available for METTL3. The metabolic balance of SAM and SAH in local subcellular environments and their control of METTL3 activity merits further investigation.

SAM binding affinity of m^6^A methyltransferase can also be regulated by substrate RNA availability. In ZCCHC4, the autoinhibitory loop interacts with the SAM-binding loop in the catalytic site, creating a closed conformation of the SAM-binding pocket [43]. This interaction is released upon 28S rRNA binding. Disruption of this intramolecular interaction by a point mutation of the autoinhibitory loop increases SAM binding affinity by four-fold, from *K*_d_ (dissociation constant) 6.7 to 1.6 µM [43]. Considering that rRNA synthesis is promoted by growth factor and nutrient-activated signaling pathways [54,55,56], it is possible that in growth-promoting conditions, increased substrate (rRNA) and methyl donor (SAM) levels cooperate for maximal rRNA methylation.

In contrast to ZCCHC4, the activity of METTL16 inversely correlates with substrate RNA binding affinity [57,58]. It has long been observed that the stability of *MAT2A* mRNA, which encodes SAM synthase, is increased by methionine depletion, while decreased in methionine-repleted conditions [59,60]. Pendleton et al. [57] and Shima et al. [58] defined a mechanism for methionine and the SAM-dependent regulation of MAT2A expression. When intracellular SAM levels are high, METTL16 actively methylates *MAT2A* mRNA and dissociates from its substrate. The m^6^A-modified *MAT2A*, which contains retained introns, is then degraded. When SAM levels are low, METTL16 tightly binds to *MAT2A* (without methylation) which leads to the efficient splicing of *MAT2A*. The spliced *MAT2A* mRNA is then translated into MAT2A protein, which synthesizes SAM [57,58] (Figure 1B). Therefore, SAM levels dictate METLL16 activity to exert the negative feedback regulation of de novo SAM synthesis, achieving a fine tuning of intracellular SAM levels. Whether other similar crosstalk exist between m^6^A enzymes and one-carbon metabolites remains unknown.

## 4. Metabolites Affecting m^6^A Erasers

### 4.1. TCA Cycle Metabolites

The demethylation of histones and DNA is dynamically regulated by various intracellular metabolites [51,61,62]. Likewise, metabolites also influence FTO and ALKBH5-mediated m^6^A RNA demethylation (Figure 2A). One example is 2-oxoglutarate (2OG, also known as alpha-ketoglutarate or αKG), the key metabolite in the citric acid cycle (tricarboxylic acid cycle, or TCA cycle) (Figure 2B). The αKG-dependent dioxygenase family proteins, which FTO and ALKBH5 belong to, require αKG, Fe(II) (non-heme iron), and O_2_ (molecular oxygen) for their full enzymatic activity [63,64]. Indeed, when αKG and iron binding sites in the αKG-Fe(II) oxygenase domain are mutated (Figure 1D), demethylation activities of FTO and ALKBH5 are lost [65,66].

In addition to αKG, the TCA cycle produces other metabolites that affect m^6^A demethylase activity (Figure 2B). αKG is oxidized and decarboxylated to produce succinate, which is further converted into fumarate. The molecular structures of succinate and fumarate are quite similar to αKG, which makes these metabolites binding competitors of αKG and thus inhibitors of m^6^A demethylases. However, only high concentrations of succinate and fumarate can inhibit αKG binding. In vitro, *K*_m_ of αKG for ALKBH5 and FTO are 2~3 µM [50,67], whereas the IC_50_ of succinate and fumarate are ~30 µM (ALKBH5) and ~150 µM (FTO), respectively [65,68]. Interestingly, another key TCA cycle metabolite, citrate, was found to occupy an αKG-binding site in ALKBH5 [65]. Citrate can also be located in the αKG-binding pocket of FTO and inhibits FTO activity with IC_50_ ~300 µM [68].

While TCA cycle metabolites are highly compartmentalized in the mitochondria, ALKBH5 and FTO are predominantly localized in the nucleus [69,70], which may hinder TCA cycle metabolite’s influence on the m^6^A demethylation process. However, there is direct evidence that TCA cycle metabolites affect FTO activity in cells. *R*-2-hydroxyglutarate (*R*-2HG) is an oncometabolite produced by cancer-associated isocitrate dehydrogenase (IDH) mutants [71]. Wild type IDH catalyzes the oxidative decarboxylation of isocitrate to αKG. In contrast, mutant IDH enzymes convert αKG to *R*-2HG. *R*-2HG has been shown to structurally mimic αKG and competitively inhibit αKG-dependent dioxygenases [72]. The IC_50_ of *R*-2HG for in vitro FTO activity is ~130 µM [73]. Cellular levels of *R*-2HG in IDH wild-type cancer cells are less than 100 µM [74], while IDH mutants increase *R*-2HG levels up to ~1000 fold in cell lines and patients [75,76,77]. The treatment of *R*-2HG (~300 µM) or ectopic expression of IDH mutants increased cellular m^6^A levels [73,75]. Surprisingly, *R*-2HG suppressed the growth of tumors expressing high FTO levels. Specifically, the *R*-2HG-induced m^6^A modification of *cMyc* and *CEBPA* mRNAs destabilized these transcripts. Therefore, by decreasing the growth-promoting cMyc and CEBP signaling activities, *R*-2HG suppresses tumor progression [73]. This anti-tumor activity of *R*-2HG was unexpected and the opposite of its oncometabolite, tumor-initiating function. This example reflects the complex nature of metabolite-mediated regulation of cellular processes and emphasizes the importance of elucidating context-dependent metabolite effects, including the unexplored area of m^6^A modifications.

### 4.2. Iron

The activation of oxygen by iron is essential for the oxidative demethylation reaction by αKG-Fe(II)-dependent dioxygenases [63,64] (Figure 2A). Indeed, iron depletion in mice and cells by diet alternation and iron chelation led to decreased histone demethylase activity [78,79]. The *K*_m_ of Fe(II) for ALKBH5 is ~1 µM [50]. This is within the range of free cellular Fe(II) (1~3 µM) [80], indicating that perturbations in cellular iron levels may affect m^6^A modification. Major organelles regulating iron metabolism are the lysosome and mitochondria [81] (Figure 2B). In mammals, the main means of iron uptake is via the transferrin–iron complex. The internalized transferrin–iron complex is delivered to the lysosome through endocytosis pathways where iron is then liberated from transferrin by low lysosomal pH and released into the cytoplasm. Therefore, the dysregulation of lysosomal acidification can potentially decrease m^6^A demethylase activity. Although there is no such direct study, iron-dependent ribosome recycling has been shown to decrease the expression of m^6^A-containing mRNAs [82]. Once released into the cytoplasm, free iron is transported into mitochondria through mitoferrin transporters [81]. Mitochondria consumes lots of iron in the production of iron–sulfur clusters and reactive oxygen species, and thus their dysfunction impairs iron homeostasis. Future investigations about how lysosomal and mitochondrial iron metabolism influences m^6^A RNA modification will provide insights not only for m^6^A metabolism but also for iron deficiency-induced human diseases.

### 4.3. NADP(H)

In a recent study, Wang et al. found that nicotinamide adenine dinucleotide (NAD) and nicotinamide adenine dinucleotide phosphate (NADP) increase FTO activity [83] (Figure 2A). Using the florescence quenching assay of FTO, they screened metabolites that directly bind to FTO. From the screen, NADH and NADPH were identified, along with vitamin C (ascorbate), a previously known cofactor of dioxygenases. Although the NAD derivatives (NAD^+^, NADH, NADP^+^ and NADPH) are structurally similar, NADPH was the strongest binding partner and activator of FTO, followed by NADH. This indicates that the reducing potential of NADPH and NADH may be used for demethylation reactions. Nonetheless, NADPH was not consumed by FTO, and the concentration remained constant during demethylation. Interestingly, the induction of m^6^A demethylase activity by NADPH occurred less in ALKBH5 (~30% induction in ALKBH5 vs. ~90% induction in FTO). Further mechanistic studies will be required to better understand the underlying mechanisms of NADPH-dependent activation of m^6^A demethylases.

The pentose phosphate pathway (PPP) is the major source of NADPH [84] (Figure 2B). Branched from glycolysis, PPP uses glucose-6-phosphate (G6P) as a primary substrate. G6P dehydrogenase (G6PD), the rate limiting enzyme in PPP, oxidizes G6P into 6-phosphogluconolactone while reducing NADP^+^ to NADPH. NADPH is a key reducing agent for cellular biosynthetic processes, such as fat synthesis. The knockdown of *NAD kinase* (*NADK*) and *G6PD* increased cellular m^6^A levels, which was decreased by NADPH supplementation. Conversely, the induction of NADPH levels by high-fat diet or glucose injection, decreased m^6^A levels [83], indicating that FTO-dependent m^6^A demethylation may be involved in the biological processes regulated by NADPH.

Indeed, the inhibition of FTO increased the m^6^A modification of the genes involved in adipocyte differentiation and blocked NADPH-induced adipogenesis [83]. *Fto* knockout mice are resistant to high-fat diet-induced obesity, while the overexpression of Fto results in obesity [83,85,86,87]. Given that *FTO* polymorphism is associated with various human metabolic diseases, including obesity, diabetes, and cardiovascular disease [88,89], it will be interesting to study how FTO and NADPH-dependent m^6^A demethylation contributes to metabolic processes in normal and pathological conditions.

## 5. Conclusions Remarks and Future Directions

Emerging evidence has implied the involvement of metabolites and metabolic pathways in m^6^A RNA modification. To better understand this important interplay in physiological and pathological contexts, more investigations are needed at the organismal level. For example, methionine is the key amino acid for SAM production. It will be interesting if a low methionine diet, which increases life span and enhances cancer treatment responses [90,91], works by decreasing the activity of specific m^6^A RNA methyltransferases. Additionally, it has been shown that m^6^A levels are different in various tissues. In mice, the brain, liver, and kidney contain more m^6^A than heart and lung. However, the expression levels of m^6^A writers and erasers only partially correlate with tissue-specific m^6^A levels [10,92]. It is possible that the metabolic activities of each organ determine the actual enzyme activities by limiting substrate and cofactor levels.

In addition to directly responding to nutrient levels, the activity of metabolic pathways is also governed by signal transduction pathways. As a master regulator of cell growth, the mechanistic target of rapamycin (mTORC1) controls the expression and activity of numerous enzymes in the metabolic pathways discussed in this review [93,94,95]. The great strides in cancer metabolism research over the past few decades have also elucidated a direct and close connection between metabolic enzymes and nutrient signaling pathways, including phosphoinositide 3-kinase (PI3K)-Akt, Ras-ERK, and AMPK [96,97,98,99]. It will be exciting to explore how these nutrient-signaling networks regulate m^6^A RNA methylation. Given that several small molecule inhibitors for m^6^A enzymes have been developed for oncological applications [100], the combined targeting of cancer metabolism and signaling with m^6^A modification enzymes could provide a new strategy for cancer therapeutics.

Another unexplored area is the metabolic regulation of m^6^A readers. The m^6^A writer, METTL3, possesses m^6^A reader function [101]. While it writes m^6^A in the nucleus, in the cytoplasm it binds to m^6^A-modified mRNA and increases target mRNA’s translation efficiency. Whether SAM or SAH, the metabolites that affect METTL3′s m^6^A writer function, can also influence METTL3′s reader function is not known. Intriguingly, some m^6^A reader proteins, specifically the YTHDF family, form liquid droplets through phase separation [46], which is also often formed by metabolic enzymes [102,103]. This implicates a potential interaction between metabolic enzymes and m^6^A readers by physical proximity. Protein interactome analysis of m^6^A readers, as well as protein–metabolite interaction screens, such as cellular thermal shift assay (CETSA) [104] and drug affinity responsive target stability (DARTS) [105], will provide useful information to identify new competitive and allosteric regulators of m^6^A readers. Since readers are actual effector proteins that determine the fates of m^6^A-modified RNAs, m^6^A reader proteins can be a way of controlling specific genes using metabolites.

In addition to m^6^A, RNA contains more than 100 different types of chemical modifications, including di- and tri-methylations, acetylation, deamination, thiolation (sulfuration), oxidation, and even glycosylation [106], which should be tightly regulated under dynamic environmental changes and cell status. Comprehensive understanding of how nutrients and metabolic pathways orchestrate the diverse array of RNA chemical modifications will provide new insights in the field of RNA epitranscriptomics, nutrient signaling, and metabolism.
